# Signature pattern of gene expression and signaling pathway in premature diabetic patients uncover their correlation to early age coronary heart disease

**DOI:** 10.1186/s13098-022-00878-x

**Published:** 2022-07-29

**Authors:** Salma Ahmadloo, King-Hwa Ling, Ahmad Fazli, Ghazaleh Larijani, Nooshin Ghodsian, Sanaz Mohammadi, Naser Amini, Vahid Hosseinpour Sarmadi, Patimah Ismail

**Affiliations:** 1 Department of Biomedical Science, Faculty of Medicine and Health Sciences, Universiti Putra Malaysia, 43400 Serdang, Selangor Malaysia; 2 Vaccination Department, Pasteur Institute of Iran, Tehran, Iran; 3 Genetics and Regenerative Medicine Research Center, Faculty of Medicine and Health Sciences, Universiti Putra Malaysia, 43400 Serdang, Selangor Malaysia; 4 Department of Medicine, Faculty of Medicine and Health Sciences, Universiti Putra Malaysia, 43400 Serdang, Selangor Malaysia; 5 Department of Biology, Science and Research Branch, Islamic Azad University, Tehran, Iran; 6 Department of Biomedical Engineering, University of British Columbia, Vancouver, Canada; 7 Faculty of Biological Science and Technology, Shahid Beheshti University, Tehran, Iran; 8 Cellular and Molecular Research Center, Iran University of Medical Sciences, Tehran, Iran; 9 Institutes of Regenerative Medicine, Faculty of Advanced Technologies in Medicine, Iran University of Medical Sciences, Tehran, Iran

**Keywords:** Coronary heart disease, Type 2 diabetes mellitus, Gene expression, Signaling pathway

## Abstract

**Background:**

Coronary Heart Disease (CHD) is the leading cause of death in industrialized countries. There is currently no direct relation between CHD and type 2 diabetes mellitus (T2D), one of the major modifiable risk factors for CHD. This study was carried out for genes expression profiling of T2D associated genes to identify related biological processes/es and modulated signaling pathway/s of male subjects with CHD.

**Method:**

the subjects were divided into four groups based on their disease, including control, type 2 diabetes mellitus (T2D), CHD, and CHD + T2D groups. The RNA was extracted from their blood, and RT^2^ Profiler™ PCR Array was utilized to determine gene profiling between groups. Finally, the PCR Array results were validated by using Q-RT-PCR in a more extensive and independent population.

**Result:**

PCR Array results revealed that the T2D and T2D + CHD groups shared 11 genes significantly up-regulated in both groups. Further analysis showed that the mRNA levels of AKT2, IL12B, IL6, IRS1, IRS2, MAPK14, and NFKB1 increased. Consequently, the mRNA levels of AQP2, FOXP3, G6PD, and PIK3R1 declined in the T2D + CHD group compared to the T2D group. Furthermore, in silico analysis indicated 36 Gene Ontology terms and 59 signaling pathways were significantly enriched in both groups, which may be a culprit in susceptibility of diabetic patients to CHD development.

**Conclusion:**

Finally, the results revealed six genes as a hub gene in altering various biological processes and signaling pathways. The expression trend of these identified genes might be used as potential markers and diagnostic tools for the early identification of the vulnerability of T2D patients to develop premature CHD.

**Graphical Abstract:**

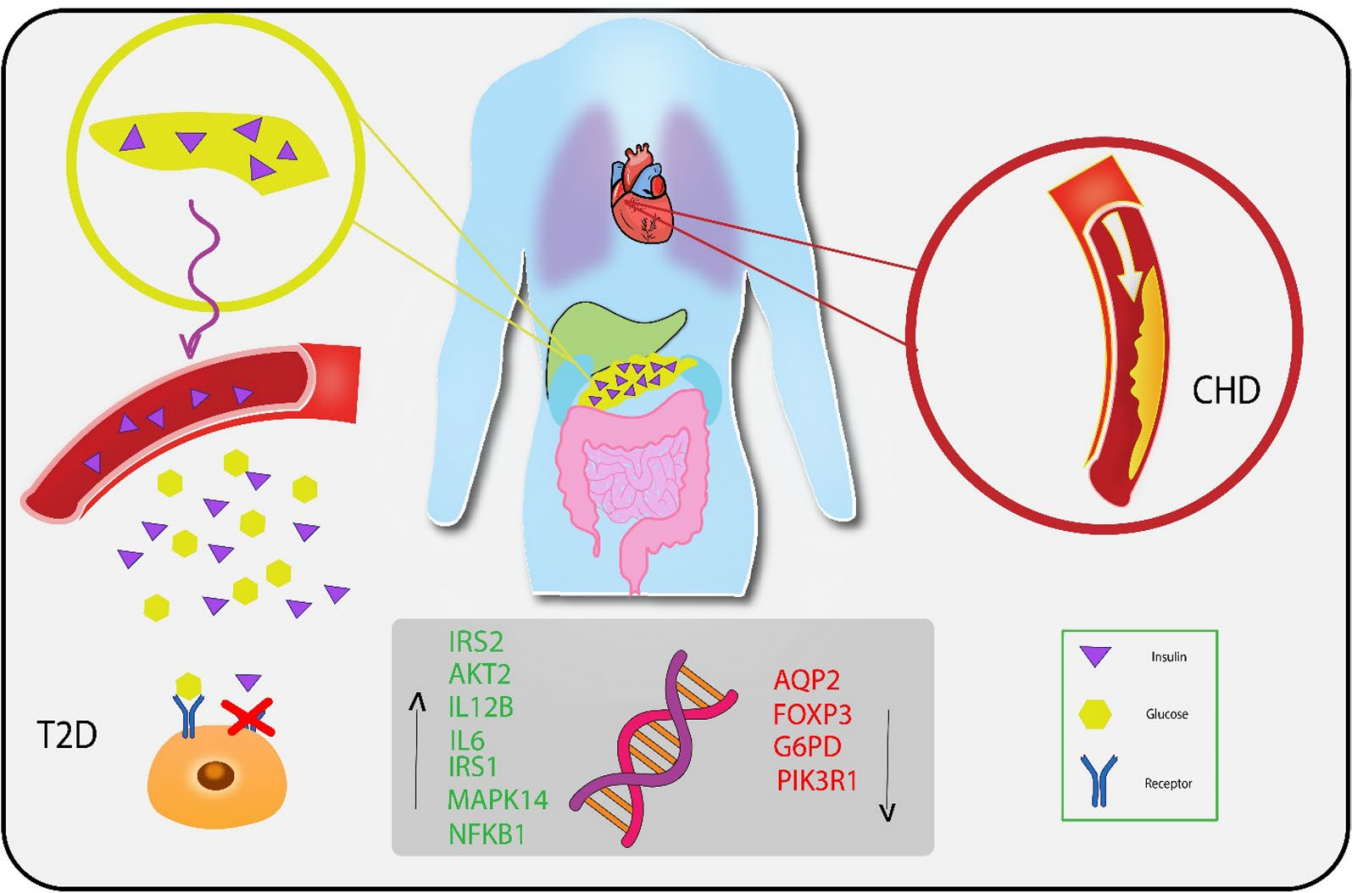

## Background

Coronary heart disease (CHD) is a common disease characterized by myocardial infarction (MI) and angina that are induced by coronary atherosclerosis, a pervasive degenerative disease in which plaques are built up in the wall of arterial vessels [1–3]. CHD is globally considered a leading cause of death in developed and developing countries [4–6]. According to the statistics issued by the American Heart Association, in 2020, ≈19 million deaths were attributed to CVD globally, which amounted to an increase of 18.7% from 2010 [7].. In Malaysia, the number of CHD patients has more than tripled in the past 40 years [8, 9]. Most CHD are thought to have a multifactorial genetic basis, involving genetic and environmental factors in interaction that determine a person’s susceptibility to the disease [10–12]. Remarkably, many of the patients with CHD have multiple risk factors, including non-modifiable such as age and gender, and changeable risk factors such as hypertension and diabetes mellitus [13–15]. Although around 80% of CHD patients are attributable to the main modifiable risk factors, the differences between the ethnic groups could be due to genetic tendencies. Gender is another important risk factor in CHD incidence, and many studies have noted differences between males and females in CHD distribution among populations [16–18]. It is well established that CHD is significantly more prevalent in the male gender [17] because the risk factors are more prevalent in males than in females. Given that CHD is increasing at an alarming rate among young males and the major risk factors are strong predictors of an increased likelihood for CHD [19, 20], there is a paucity of data regarding the mechanism of the major risk factors and CHD development. Thus, there is an urgent need to determine the association between genes related to the major risk factors and male subjects with CHD.

On the other hand, the past decade has seen the rapid development of type 2 diabetes mellitus (T2D) in many countries. Although insulin resistance and insufficient insulin secretion are essential in T2D development, genetic defects predisposed to both are likely to be important contributors to the disease process [21, 22]. It was shown that the mortality in T2D was mainly driven by the vascular complications of CHD and stroke [23]. Furthermore, CHD and T2D are the two leading causes of death worldwide, and patients with T2D are at two to six times greater risk of developing CHD than those without T2D [24, 25].

Thus far, there has been little discussion about the genomic architectures shared by T2D and CHD because the genes and respective molecular pathway/s involved in CHD development in diabetic patients remain largely unknown. The current research is designed based on a case/control study for analyzing gene/s expression profiling related to T2D in CHD patients. This study has investigated and identified risk genes and modulated pathway/s associated with T2D, which confer risk to developing early age CHD. Therefore, the evaluation of differentially expressed genes associated with T2D and CHD is the key to understanding the contributed genes and subsequently signaling pathway/s in CHD development in T2D patients. In this regard, the co-expression genes, genes that have similar expression patterns, in T2D and T2D plus CHD were evaluated to identify co-expression gene/s and pathway/s which enriched for T2D and T2D plus CHD genetic associations.

## Methods

### Study participants

The initial sample consisted of 600 subjects in Hospital Serdang, Malaysia. A total of 108 Malaysian males belonging to 3 main ethnic groups in Malaysia, namely the Malays, Chinese, and Indians with median age 47.5 ± 7.5 years who met the inclusion and exclusion criteria and suffering from CHD and T2D or either of them were selected. Furthermore, a group of age-matched healthy controls (HCs) were recruited (36 individuals). In order to identify CHD and T2M, patients in the current study were selected based on angiographic confirmation of coronary stenosis ≥ 50% and physician diagnosis and fasting plasma glucose level ≥ 7.0 mmol/l, respectively. The recruited subjects patients were divided into four groups based on medical records, 36 subjects with T2M, 36 subjects with CHD, 36 subjects with both diseases (T2M + CHD), and 36 healthy individuals as a control group. The Ethics Committee of the Faculty of Medicine and Health Sciences, Universiti Putra Malaysia (UPM), and the National Medical Research Register (NMRR) approved the current study (23 January 2013). Furthermore, all patients gave informed consent before their inclusion into the study. The reason that only males were recruited in this study was that CHD is significantly more prevalent in the male gender (because the risk factors are more prevalent in males than in females). In addition, a woman’s reproductive status, menstrual cycle and contraceptive history have become significant interfering factors in researach studies.

### Study design

**Fig. 1:**
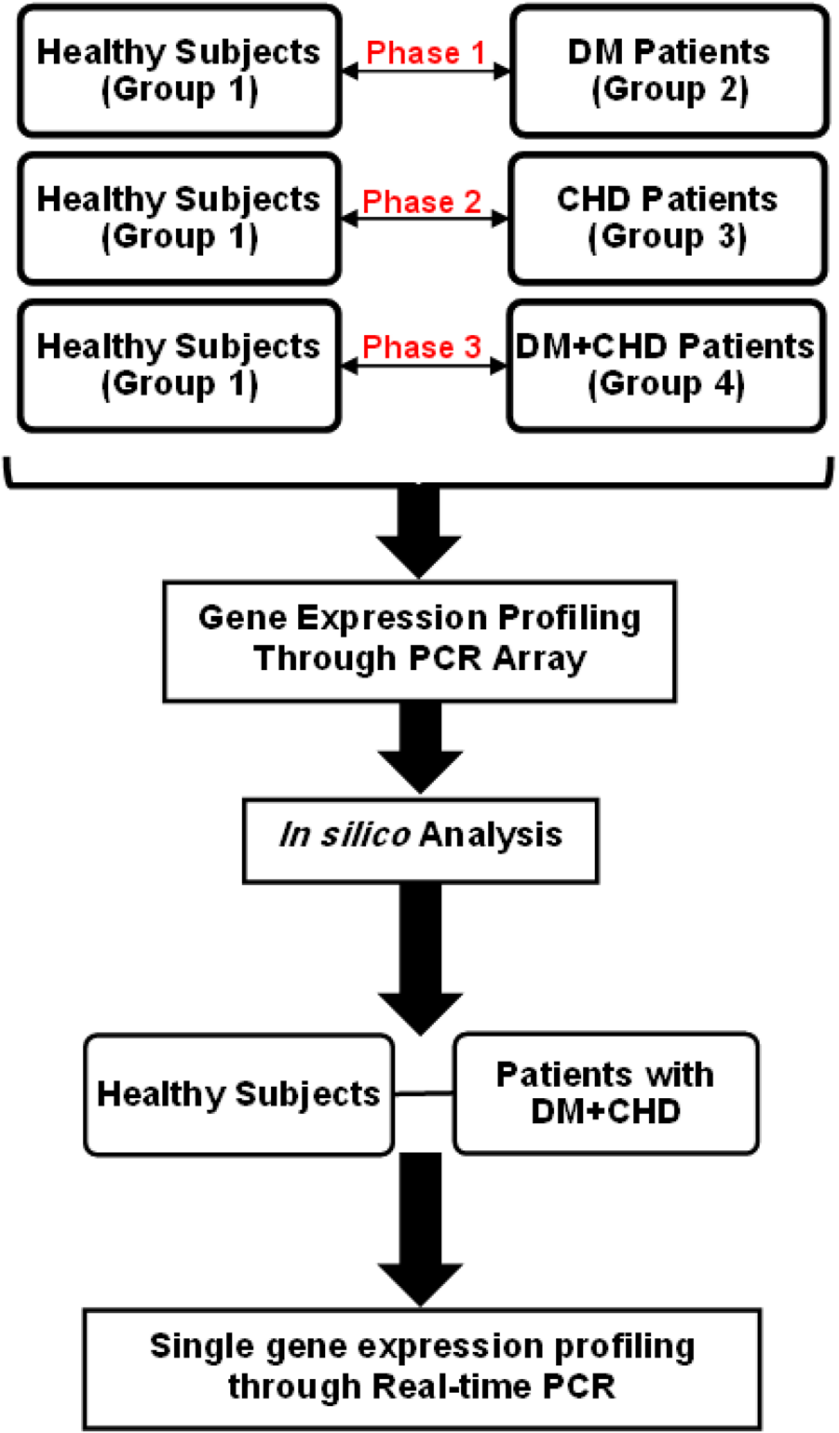
Diagram of study design

As shown in Fig. 1, gene profiling conducted through RT^2^ profiler™ PCR Array on the control group and T2M group, control group and CHD group, control group and T2D + CHD group were identified. Finally, RT^2^ profiler™ PCR Array results were validated through Q-RT-PCR in a larger and independent population. The control group was chosen among the Malaysian males with a negative record of any cardiovascular disease, acute or chronic illnesses, and impaired fasting glucose or diabetes (Fasting plasma glucose level ≥ 7.0 mmol/l). As shown in Table 1, all the subjects were asked to fill in a demographic characteristics questionnaire form, which included the following items: age, gender, weight, height, race, smoking history, marital status, and family history of CHD. Furthermore, all recruited participants were confirmed free from any late diabetic complications (such as proliferative retinopathy, consolidated nephropathy, kidney failure, heart disease, and autonomic neuropathy), influencing the results. Table 1 shows that all the subjects' main biochemical factors, including a lipid profile, fasting glucose levels, anthropometric variables, and blood pressure were recorded based on the patients’ medical records.

**Table 1:** The mean differences in continuous traits and the frequencies between all groups were tested by chi-square test

Groups	Control vs. T2D	Control vs. CHD	Control vs. T2D + CHD	T2D vs. CHD	T2D vs. T2D + CHD	CHD vs. T2D + CHD
	M(SD)P	M(SD)P	M(SD)P	M(SD)P	P	P
Factors						
Age	54(3.7)1.00	53(5.2)0.76	52(5.2)1.00	53(1.7)1.00	1.00	1.00
Weight	74(6.9)0.001	91(11.4)0.28	82(15.7)0.000	96(10.1)0.33	1.00	0.01
BMI	23(0.8)0.000	28(3.6)0.002	28(4.6)0.000	30(2.6)1.00	1.00	0.64
Glucose	4.0(0.6)0.000	9.9(1.2)1.00	3.7(0.58)0.000	8.7(0.60)0.000	0.001	0.000
Hb1Ac	4.3(0.6)0.000	8.7(1.0)1.00	4.2(0.6)0.000	8.0(0.8)0.000	0.16	0.000
WBC	6.2(0.8)1.00	6.4(0.8)1.00	6.2(0.8)0.04	7.1(1.2)1.00	0.24	0.06
HB	15(0.5)0.74	14(1.0)1.00	15(0.8)1.00	15(0.9)1.00	0.51	1.00
PLC	316(78)1.00	300(69)1.00	320(85)1.00	314(77)1.00	1.00	1.00
TG	1.4(0.4)0.36	2.0(1.2)0.38	2.0(0.7)0.21	2.1(0.8)1.00	1.00	1.00
Chol	4.1(0.6)1.00	4.3(1.6)0.03	5.4(1.2)0.02	5.5(1.3)0.11	0.07	1.00
LDL	1.2(0.1)0.007	2.4(1.1)0.16	2.0(0.6)0.000	5.1(1.5)1.00	0.000	0.000
HDL	2.9(0.4)0.00	1.4(0.3)0.003	2.0(0.6)0.04	2.2(1.0)0.09	0.008	1.00

Number of individuals in each group are 36 person and means, standard deviation and p value are indicated by M, SD and P, respectively

### RNA extraction and cDNA synthesis

Peripheral blood (3 ml) was collected from all subjects by a qualified nurse and preserved in PAXGene Tubes (QIAGEN, Germany). According to the manufacturer’s instructions, RNA isolation was performed using the PAXGene Blood RNA Kit (QIAGEN, Germany). Briefly, PAXGene blood tubes were centrifuged, and the pallet was added 5 ml of RNase-free water and, after mixing, were centrifuged at 5000*g* for 10 min. Then, to bring about protein digestion, the resuspended pellet was incubated in optimized lysis buffers and 40 µl of the proteinase K solution in the shaker incubator at 55 °C for 10 min. After centrifugation through a microcentrifuge, the supernatant was moved to a fresh 2 ml microcentrifuge tube; after that, absolute ethanol was added to adjust binding conditions and mixed by a vortex. Afterwards, the supernatant was applied to the PAXGene RNA Spin column and centrifuged for 1 min at ≥ 8000*g* to pass through contaminants. RNA was selectively bound to the PAXgene silica membrane. The PAXGene Spin column was placed in a new 2 ml processing tube, and the membrane was treated with DNase to remove trace amounts of bound DNA. Also, the column was washed with buffers supplied in the reagent set. Finally, to elute pure RNA, 40 µl of elution buffer was applied, and eluents were incubated for 5 min at 65 °C followed by immediate chilling on ice. The quantity of isolated RNA was determined by NanoDrop Technologies ND-1000 spectrophotometer (Thermo Scientific, USA), and then all samples were stored at − 80 °C for the subsequent experiments. Agilent 2100 Bioanalyzer analyzed the RNA integrity and genomic DNA contamination of samples. To cDNA synthesis, samples with high quality of RNA was used with RNA integrity number (RIN ≥ 8.0), A_260/A230_ ratio (2.0–2.2), A_260/A280_ ratio (1.8–2.0) with reasonable concentration (> 100 ng/μL). cDNA was synthesized from total RNA (400 ng) using RT^2^ First Strand Kit (QIAGEN, Cat. No. 330404) under the manufacturer’s instructions. Briefly, RNA samples were mixed with GE buffer (5 × g DNA Elimination Buffer) and RNase-Free Water and were incubated on a thermal cycler. For reverse transcript, 10 µl genomic DNA elimination mix was added into the tube containing 10 µl Reverse-transcription mixture followed by incubation at 42 °C for 15 min and then terminated with 95 °C heat for 5 min. 91 µl RNase-Free water was added to each 20 µl cDNA synthesis reaction, and the transcribed cDNA was held in a – 20 °C fridge for further experiments.

### RT^2^ profiler™ PCR array

To evaluate the gene profiling of 84 key genes related to the onset, development, and progression of T2D among Malaysian males, the Human Diabetes RT^2^ Profiler™ PCR Array (Cat. No. PAHS-023Z) was utilized. In this regard, the previously prepared cDNA was mixed with RT^2^ SYBR Green ROX FAST Mastermix and RNase-free water according to the manufacturer’s instruction. Subsequently, 20 µl of the mixture was loaded to each well of 100-well disc PCR array and then was run according to cycling program (1 cycle in 95 °C for 10 min, 40 cycles in 95 °C for 15 s, and 60 °C for 30 s). The 100-well disc PCR array consisted of: (1) 84 wells of optimized primer for each specific 84 genes. (2) Five wells contained five different housekeeping genes, which enable the normalization of data. (3) One well contained optimized primer for genomic DNA control (GDC) to specifically recognize non-transcribed genomic DNA contamination. (4) Three wells as a replicate reverse-transcription controls (RTC) primer to control the efficiency of the reverse-transcription reaction completed with the RT^2^ First Strand Kit. (5) Three wells contained pre-coated replicate positive PCR controls (PPC) primer to detect a pre-dispensed artificial DNA sequence. (6) Four empty wells were loaded with RNase-free water to make a 100-well disc balance. Finally, relative expression intensity was analyzed by Rotor-Gene Q software version 2.3.1 based on the ΔΔC_T_ method, calculating the threshold (CT) for each well. The obtained CT values were analyzed by web-based PCR Array data analysis software version 3.5 downloaded from www.SABiosciences.com/pcrarraydataanalysis.php. The obtained CT values from different samples were directly normalized to housekeeping genes and then were compared. Alterations in the mRNA expression were compared between various groups of study, and the results were reported as fold-change (FC). Fold change greater than two and p-value less than 0.05 was considered a significant change.

### Gene ontology (GO) and pathway enrichment analyses

All ordinary differentialy expressed genes between phases 1 and 3 were analyzed by DAVID (Database for Annotation, Visualization, and Integrated Discovery) online bioinformatics software. The results were assessed for GO enrichment analysis and Kyoto Encyclopedia of Genes and Genomes (KEGG) pathway analysis to annotate the dysregulated genes’ biological function and signaling pathway through the DAVID online software by 0.05 cut-off.

### Validation by real-time PCR

In order to validate the PCR array results, real-time PCR (Q-RT_PCR) was performed to analyze single gene expression. Five significantly dysregulated genes in PCR array results were selected to validate by Q-RT-PCR in an independent and larger population. In this regard, 150 individuals who met the research’s criteria were recruited; 50 control, 50 T2D, and 50 patients with diabetes who then developed CHD. The Q-RT-PCR run was performed using the RT^2^ SYBR Green Mastermixes (Cat. No. 330523, Qiagen, Hilden, Germany) by using the Rotor-Gene 6000 instruments (Corbett Life Science, Valencia, CA, and the USA). Laboratory verified RT^2^ Primer Assays (Qiagen) was used to analyze expression levels of exciting genes and housekeeping gene. The β-actin (ACTB; PPM02945A) and glyceraldehyde 3-phosphate dehydrogenase (GAPDH; PPH00150A) as constitutively active gene control were used to normalize the tested genes. The selected genes included IL6 (PPH00560C), G6PD (PPH02322H), AKT2 (PPH00289F), FOXP3 (PPH00029C), and IRS1 (PPH02328A). PCR component for each reaction (25 μl) contained 12.5 μl RT^2^ SYBR Green Mastermixes, 1 μl of RT^2^ Primer Assays, 1 μl of template cDNA, and 10.5 μl RNase free water. Afterwords, thermo cycle protocol was used for Q-RT-PCR amplification: 95 °C for 10 min, 40 cycles of 95 °C for 15 s, and 72 °C for 30 s. Individual cDNA samples were run in triplicates for each gene of interest, including endogenous controls. Each sample was run in triplicate, and averaged triplicate was used to determine Ct values. Data were normalized for the housekeeping genes, and the 2^−ΔΔCt^ method was used to determine the fold changes in gene expression.

### Statistical analysis

Statistical analyses were performed by SPSS software version 19 with values expressed as a mean ± standard deviation (SD) of three independent experiments. One-way ANOVA, an analysis of variance, was used to determine the statistically significant differences between different groups. A chi-square test was used using crosstab to compare the frequencies or proportions of categorical variables between groups. A *p*-value less than 0.05 was used to determine the statistically significant differences.

## Results

### General descriptive results

In order to understand the mechanisms underlying CHD pathogenesis factors, relations between sociodemographic with CHD development were taken into account. Thus, an attempt was made to find the differences in sociodemographic and clinical characteristics associated with CHD and T2D to capture the indirect effects of these factors on the selected diseases. To this end, requited data were categorized into continuous and categorical factors and SPSS ver. 19.0 were used for statistical analysis. As shown by Tables 1 and 2, the p values in stable traits and the frequencies or proportions of categorical variables between all groups were tested by One-way ANOVA and chi-square test, respectively. Values are expressed as mean ± SD for all continuous variables. A *p*-value ≤ 0.05 was considered statistically significant.

**Table 2:** The mean differences in frequencies and the proportions of categorical variables between all groups were tested by chi-square test

Groups	Control vs. T2D (*p*-value)	Control vs. CHD (*p*-value)	Control vs. T2D + CHD (*p*-value)	T2D vs. CHD (*p*-value)	T2D vs. T2D + CHD (*p*-value)	CHD vs. T2D + CHD (*p*-value)
Variable						
Smoking	0.17	0.06	0.04	0.36	0.63	0.69
Race	0.64	0.12	0.24	0.30	0.67	0.58
Family history	0.62	0.008	0.003	0.02	0.01	0.70

Number of individuals in each group are 36 person

The results analysis showed that the average age for the controls group was 54 and for patient groups were as follows: T2D (53), CHD (52), and T2D + CHD (53). Consequently, the different comparison of the mean age of both groups revealed no statistically significant differences between the groups: control, T2D, CHD, and T2D + CHD. On the contrary, the results of weight and BMI illustrated a statistically significant difference between patients suffering from T2D (p = 0.00) and, consequently, patients with having both conditions (T2D + CHD) (p = 0.00) compared to the control group. In parallel, control vs. CHD (p = 0.002) comparison was found to be significant. The classification of overweight and obesity is based on measuring the BMI calculated in the metric system as the ratio of weight in kilogram to the square of the height in meter. A BMI more than 25 is defined as overweight, and a BMI ≥ 30 is regarded as obesity [26]. Moreover, fasting blood glucose and HbA1c as two main clinical factors in diabetic patients were compared to find significant differences between the groups under the study. The compression showed a significant difference between the groups with T2D compared to other groups, as expected (Control vs. T2D, p = 0.00) (Control vs. T2D + CHD, p = 0.00). In addition, the lipid profile evaluation (total cholesterol, LDL, HDL, and TG) showed significant differences in all lipid factors except TG between the patients of groups with T2D, CHD, and T2D + CHD against the control group with an exception in cholesterol and LDL factors in the control group compared to T2D and CHD, respectively. In parallel, although white blood cells (WBCs) were slightly higher in all patients’ groups against the control group, a significant difference was founded only in patients with T2D + CHD compared to healthy people (Control vs. T2D + CHD, p = 0.04). Furthermore, the results revealed no significant differences between the understudy groups regarding Platelets (PLC) and Hemoglobin (HB).

In the current study, smoking status was defined as present, never, and former user (no cigarettes within the past 30 days). The results showed significant differences between patients with CHD and T2D + CHD compared to the healthy group (Control vs. T2D + CHD, p = 0.04), as expected. Furthermore, the race evaluation in current research illustrated no significant differences between the subjects’ race between the groups. However, the number of ethnic Malays was predominant in all subsets of patients and controls. Finally, the family history in the current research was defined as at least one first-degree relative (parents/siblings) diagnosed with CHD. As anticipated, the majority of the subjects in the groups with CHD and T2D + CHD group had a positive family history compared to the healthy groups (Control vs. CHD, p = 0.008) (Control vs. T2D + CHD, p = 0.003). The statistical analysis showed that there was a strong association between family history and early age CHD since all of the patients with CHD had developed the disease before 60 based on their medical records in hospital Serdang, confirming the results from numerous studies.

### Expression profiling between T2D, CHD, and T2D + CHD subjects vs. unaffected subjects

To identify the specific responsible risk genes for T2D (phase 1) that trigger risk to developing CHD, first of all, the expression pattern of the selected genes as T2D risk genes in the Malaysian population, where the research was conducted, were evaluated by profiling of 84 genes in the patients with T2D only compared to the healthy control group to screen their pattern. As shown in Table 3, the gene profiling results revealed that 36 of 84 genes were dysregulated in a Malaysian population, that 30 and 6 genes were up and down-regulated in Malaysian diabetic patients compared to healthy people. As Table 4 illustrates, to filter the genes that independently are risk genes for CHD development in the Malaysian population (phase 2), profiling of gene expression was performed between Malaysians suffering from only CHD compared to healthy controls. The results showed 10 dysregulated genes whose expression levels were statistically different. In the third group, to identify specific T2D related genes which predispose diabetic subjects to CHD development (phase 3), gene expression profiling was performed in diabetic patients who later (at least 5 years) had developed CHD (based on the ages at the time of diagnosis in their medical records in hospital Serdang) in comparison with the control group as can be seen from the Table 5 gene expression profiling identified 21 genes, which were significantly dysregulated between these groups.

**Table 3:** List of significantly dysregulated genes in T2D vs. control groups

Symbol	Description	Fold change	*p*-value
ABCC8	ATP-binding cassette	↑50.7	0.00
ADRB3	Adrenergic, beta-3-, receptor	↑203.1	0.03
AGT	Angiotensinogen (serpin peptidase inhibitor)	↑10.67	0.008
AKT2	V-akt murine thymoma viral oncoGene homolog 2	↑120.8	0.027
AQP2	Aquaporin 2 (collecting duct)	↑456.1	0.000
CTLA4	Cytotoxic T-lymphocyte-associated protein 4	↑683.4	0.002
ENPP1	Ectonucleotide pyrophosphatase/phosphodiesterase 1	↑215.2	0.030
FBP1	Fructose-1,6-bisphosphatase 1	↑456.1	0.047
FOXC2	Forkhead box C2 (MFH-1, mesenchyme forkhead 1)	↑608.8	0.032
FOXP3	Forkhead box P3	↑683.438	0.005
G6PD	Glucose-6-phosphate dehydrogenase	↑16,007.9	0.009
GCGR	Glucagon receptor	↑95.8917	0.044
GLP1R	Glucagon-like peptide 1 receptor	↓0	0.027
GSK3B	Glycogen synthase kinase 3 beta	↓0	0.004
HMOX1	Heme oxygenase (decycling) 1	↓0.00	0.002
HNF1B	HNF1 homeobox B	↓0.00	0.007
ICAM1	Intercellular adhesion molecule 1	↓0.00	0.000
IDE	Insulin-degrading enzyme	↓0.002	0.00
IFNG	Interferon, gamma	↑287.3	0.014
IL10	Interleukin 10	↑1366.8	0.005
IL12B	Interleukin 12B (natural killer cell stimulatory factor 2)	↑362.03	0.04
IL4R	Interleukin 4 receptor	↑85.4	0.04
IL6	Interleukin 6 (interferon, beta 2)	↑1149.4	0.00
INSR	Insulin receptor	↑228.07	0.03
IRS1	Insulin receptor substrate 1	↑120.81	0.03
IRS2	Insulin receptor substrate 2	↑120.81	0.030
MAPK14	Mitogen-activated protein kinase 14	↑1084.8	0.046
ME1	Malic enzyme 1, NADP (+)-dependent, cytosolic	↑152.21	0.006
NFKB1	Nuclear factor of kappa light polypeptide Gene enhancer in B-cells 1	↑1824.5	0.026
PDX1	Pancreatic and duodenal homeobox 1	↑8	0.039
PIK3R1	Phosphoinositide-3-kinase, regulatory subunit 1 (alpha)	↑6501.9947	0.038
PPARGC1B	Peroxisome proliferator-activated receptor gamma, coactivator 1 beta	↑107.6347	0.007
PRKAG2	Protein kinase, AMP-activated, gamma 2 non-catalytic subunit	↑574.7006	0.016
RAB4A	RAB4A, member RAS oncoGene family	↑3250.9974	0.001
RETN	Resistin	↑7732.2184	0.000
SLC2A4	Solute carrier family 2 (facilitated glucose transporter), member 4	↓0.7492	0.042

The symbol ↑ and ↓ reflect the genes up and down-regulation, respectively

**Table 4:** Significantly dysregulated genes in CHD vs. control groups

Symbol	Description	Fold change	*p*-value
CCR2	Chemokine (C–C motif) receptor 2	↑8847.8494	0.032528
FOXG1	Forkhead box G1	↑620.7109	0.04413
G6PC	Glucose-6-phosphatase, catalytic subunit	↑1970.6344	0.02753
GCK	Glucokinase (hexokinase 4)	↑260.9627	0.041348
GPD1	Glycerol-3-phosphate dehydrogenase 1	↓0.0084	0.024563
IDE	Insulin-degrading enzyme	↓0.0253	0.003735
IFNG	Interferon, gamma	↑184.5385	0.041682
IL4R	Interleukin 4 receptor	↑4.0778	0.036172
NEUROD1	Neurogenic differentiation 1	↑4087.7017	0.024778
PRKAG2	Protein kinase, AMP-activated, gamma 2	↑1765.0753	0.036329

The symbol ↑ and ↓ reflect the genes up and down-regulation, respectively

**Table 5:** Significantly dysregulated genes in T2D + CHD vs. control groups

Symbol	Description	Fold change	*p*-value
ACLY	ATP citrate lyase	↑620.7109	0.008527
AKT2	V-akt murine thymoma	↑2786.8979	0.031769
AQP2	Aquaporin 2	↑8.1555	0.045277
FOXP3	Forkhead box P3	↑3.8489	0.047164
G6PD	Glucose-6-phosphate dehydrogenase	↑13,256.7953	0.01726
GLP1R	Glucagon-like peptide 1 receptor	↓0.0002	0.005732
GPD1	Glycerol-3-phosphate dehydrogenase 1 (soluble)	↓0.003	0.022912
GSK3B	Glycogen synthase kinase 3 beta	↓0.0032	0.010075
HMOX1	Heme oxygenase (decycling) 1	↓0.0008	0.007296
ICAM1	Intercellular adhesion molecule 1	↓0.0001	0.001424
IDE	Insulin-degrading enzyme	↓0.0063	0.003091
IKBKB	Inhibitor of kappa light polypeptide gene enhancer in B-cells	↑438.9089	0.0001
IL12B	Interleukin 12B (natural killer cell stimulatory factor	↑877.8178	0.040432
IL6	Interleukin 6 (interferon, beta 2)	↑1970.6344	0.011704
IRS1	Insulin receptor substrate 1	↑2482.8438	0.034903
IRS2	Insulin receptor substrate 2	↑828.5497	0.000069
MAPK14	Mitogen-activated protein kinase 14	↑2630.4814	0.015893
NFKB1	Nuclear factor of kappa light polypeptide Gene enhancer in B-cells 1	↑4686.9849	0.006946
PIK3R1	Phosphoinositide-3-kinase, regulatory subunit 1	↑1315.2407	0.021226
STX4	Syntaxin 4	↓0.0003	0.00881
TRIB3	Tribbles homolog 3 (Drosophila)	↓0.0084	0.044704

The symbol ↑ and ↓ reflect the genes up and down-regulation, respectively

**Fig. 2:**
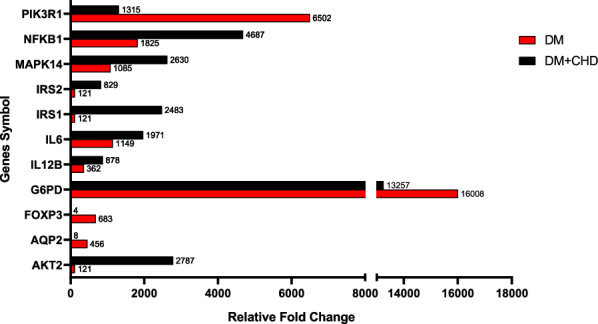
11 dysregulated diabetes-related genes as target genes. *IL12B* (interleukin-12 subunit beta), *IL6* (interleukin 6), *IRS* (insulin receptor substrate), *MAPK14* (mitogen-activated protein kinase 14), *NFKB1* (nuclear factor NF-kappa-B subunit 1), *AQP2* (aquaporin 2), *FOXP3* (Forkhead box P3), *G6PD* (glucose-6-phosphate dehydrogenase), *PIK3R1* (phosphoinositide-3-kinase regulatory subunit 1)

Finally, to achieve the proper genes which confer risk to CHD development, filtering the genes that independently are risk genes for CHD development in the Malaysian population was a must. Thus, the significantly dysregulated genes in phase 2 were ignored. The commonly dysregulated genes between phases 1 and 3 with minimum two-fold differential expression and p-value < 0.05 were used as a cut-off for inclusion into further analysis. Considering the criteria mentioned above, 11 dysregulated diabetes-related genes were identified which may predispose to CHD development are shown in Fig. 2. Interestingly, all the identified 11 genes were up-regulated in both phases, especially G6PD, which severely rose in both groups compared to the control group. Furthermore, more analysis revealed that some of these up-regulated genes dramatically increased or decreased in T2D plus CHD group compared to T2D only groups, such as FOXP3 and AQP2 decreased 170 and 57 times, respectively, or AKT2 and IRS1 increased more than 20 times compared to T2D group.

### Gene ontology analysis

To evaluate the potentially altered biological process related to differentially expressed genes in the final generated genes list, the gene ontology (GO) analysis was performed. To this end, the final 11 dysregulated genes with two-fold change and p-value < 0.05 as cut off were analyzed by DAVID functional annotation tool [27]. The degree of enrichment that statistical methods can quantify includes the EASE score p-value and the Fisher’s Exact test. GO enrichment analysis of the 11 dysregulated genes by DAVID bioinformatics tools revealed that 36 GO terms were significantly enriched with an EASE score *p*-value less than 0.05. Table 6 shows the top 20 significant enriched functional categories. Positive regulation of glucose import, glucose metabolic process, cellular response to insulin stimulus, and insulin receptor signaling pathway are the most significantly enriched biological processes in T2D and T2D with CHD groups. Experimental evidence in the published literature is the base of the GO clustering system, and each gene product can have one or more biological processes. In this regard, further analysis of differentially expressed genes in 36 biological processes illustrated that PIK3R1 (47.2%), IRS1 (44.4%), IRS 2 (41.7%), and AKT2 (33.3%) were the most common genes in various overrepresented functional categories.

**Table 6:** Top twenty overrepresented biological process

Term	Count	p-value	Gene symbol
Positive regulation of glucose import	5	1.70E−09	AKT2, IRS1, IRS2, MAPK14, PIK3R1
Glucose metabolic process	4	7.10E−06	AKT2, G6PD, IRS2, MAPK14,
Cellular response to insulin stimulus	4	1.10E−05	AKT2, IRS1, IRS2, PIK3R1
Insulin receptor signaling pathway	4	1.10E−05	AKT2, IRS1, IRS2, PIK3R1
Positive regulation of fatty acid beta-oxidation	3	1.10E−05	AKT2, IRS1, IRS2
Positive regulation of glucose metabolic process	3	1.10E−05	AKT2, IRS1, IRS2
Positive regulation of glucose import in response to insulin stimulus	3	2.50E−05	AKT2, IRS1, PIK3R1
Positive regulation of glycogen biosynthetic process	3	3.30E−05	AKT2, IRS1, IRS2
Cellular response to lipopolysaccharide	4	3.40E−05	IL12B, IL6, MAPK14, NFKB1
Signal transduction	6	2.90E−04	AKT2, IRS1, IRS2, MAPK14, NFKB1, PIK3R1
Phosphatidylinositol-3-phosphate biosynthetic process	3	3.70E−04	IRS1, IRS2, PIK3R1
Regulation of phosphatidylinositol 3-kinase signaling	3	9.40E−04	IRS1, IRS2, PIK3R1
Phosphatidylinositol phosphorylation	3	1.40E−03	IRS1, IRS2, PIK3R1
Phosphatidylinositol-mediated signaling	3	1.70E−03	IRS1, IRS2, PIK3R1
Positive regulation of transcription from RNA polymerase II promoter	5	1.80E−03	FOXP3, IL6, MAPK14, NFKB1, PIK3R1
Negative regulation of plasma membrane long-chain fatty acid transport	2	2.40E−03	AKT2, IRS2
T cell receptor signaling pathway	3	3.30E−03	FOXP3, NFKB1, PIK3R1
Positive regulation of cell migration	3	5.10E−03	AKT2, IRS2, PIK3R1
Negative regulation of interleukin-17 production	2	6.50E−03	FOXP3, IL12B
Negative regulation of interleukin-10 production	2	7.10E−03	FOXP3, IL12B

### Pathways analysis

In order to uncover putative cellular signaling pathway/s associated with the final 11 up-regulated genes, the DAVID bioinformatics tool was used to map genes to pathways. Kyoto Encyclopedia of Genes and Genomes (KEGG) pathway was utilized through DAVID resource to find the significant pathway/s based on p-value (p < 0.05). Data from Table 7 shows that 59 signaling pathways were significantly enriched in both phases mentioned above, indicating the top twenty significantly overrepresented signaling pathways by up-regulated genes. Toll-like receptor signaling pathway, insulin resistance, and FoxO signaling pathway were the most significant enriched signaling pathway. Furthermore, the results deciphered that AKT2 (81.4%), PIK3R1 76.3%), NFKB1 (66.1%), and MAPK14 (47.5%) were the most interferer genes in the known different signaling pathways.

**Table 7:** Top 20 signaling pathway mediated by the identified genes

Term	Count	p-value	Gene symbol
Toll-like receptor signaling pathway	6	1.90E−07	AKT2, IL12B, IL6, MAPK14, NFKB1, PIK3R1
Insulin resistance	6	2.10E−07	AKT2, IRS1, IRS2, IL6, NFKB1, PIK3R1
FoxO signaling pathway	6	6.10E−07	AKT2, IRS1, IRS2, IL6, MAPK14, PIK3R1
Non-alcoholic fatty liver disease (NAFLD)	6	1.10E−06	AKT2, IRS1, IRS2, IL6, NFKB1, PIK3R1
Influenza A	6	2.20E−06	AKT2, IL12B, IL6, MAPK14, NFKB1, PIK3R1
TNF signaling pathway	5	1.10E−05	AKT2, IL6, MAPK14, NFKB1, PIK3R1
Neurotrophin signaling pathway	5	1.70E−05	AKT2, IRS1, MAPK14, NFKB1, PIK3R1
Measles	5	2.60E−05	AKT2, IL12B, IL6, NFKB1, PIK3R1
Regulation of lipolysis in adipocytes	4	5.90E−05	AKT2, IRS1, IRS2, PIK3R1
Tuberculosis	5	7.90E−05	AKT2, IL12B, IL6, MAPK14, NFKB1
Inflammatory bowel disease (IBD)	4	8.80E−05	FOXP3, IL12B, IL6, NFKB1
Adipocytokine signaling pathway	4	1.20E−04	AKT2, IRS1, IRS2, NFKB1
Prolactin signaling pathway	4	1.20E−04	AKT2, MAPK14, NFKB1, PIK3R1
Pertussis	4	1.40E−04	IL12B, IL6, MAPK14, NFKB1
HIF-1 signaling pathway	4	2.90E−04	AKT2, IL6, NFKB1, PIK3R1
T cell receptor signaling pathway	4	3.30E−04	AKT2, MAPK14, NFKB1, PIK3R1
Amoebiasis	4	3.90E−04	IL12B, IL6, NFKB1, PIK3R1
Toxoplasmosis	4	4.40E−04	AKT2, IL12B, MAPK14, NFKB1
Sphingolipid signaling pathway	4	5.70E−04	AKT2, MAPK14, NFKB1, PIK3R1
AMPK signaling pathway	4	6.10E−04	AKT2, IRS1, IRS2, PIK3R1

### Validation of PCR array’s results by Q-RT-PCR

**Fig. 3:**
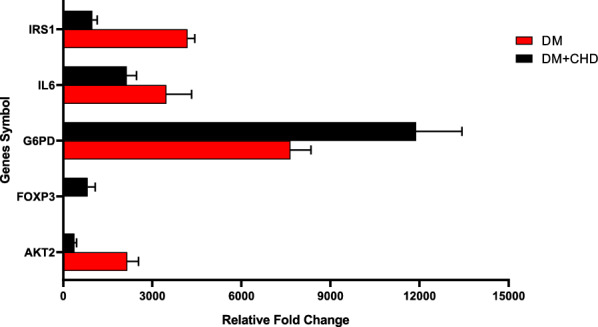
The Q-RT-PCR results revealed a consistent expression pattern that was found in PCR array for all five genes examined. *IL6* (interleukin 6), *IRS* (insulin receptor substrate), *FOXP3* (forkhead box P3), *G6PD* (glucose-6-phosphate dehydrogenase)

In order to confirm the PCR array’s results, additional analyses by using Q-RT-PCR were utilized. In this regard, among the 11 genes, five genes were randomly selected (G6PD, IRS1, AKT2, FOXP3, and IL-6) for Q-RT-PCR analysis by using SYBR Green. The data in Fig. 3 indicates that the Q-RT-PCR results revealed a consistent expression pattern found in the PCR array for all five genes examined.

## Discussion

Numerous studies have claimed that sociodemographic factors such as age, weight, BMI, blood glucose, lipid profile, smoking status, etc., strongly affect CVDs and influence all of the CVDs risk factors [28, 29]. In the current study, the mean age comparison between groups revealed no significant differences among the groups. Due to the biological aging process of organs and cells in the human body, CVDs are mainly experienced in older ages without reference to the genetic background [30, 31]. It was shown that the development of atherosclerosis and T2D is a long-term process. Still, the genetic heritage could accelerate these processes, meaning that these conditions do not generally appear at young ages [32]. However, the weight and BMI results showed significant differences between T2D and coexisting diseases (T2D + CHD) compared to the control group. Around 90–95% of diagnosed diabetes in adults are obesity-associated diabetes [33–35]. Several studies have shown that obesity is extensively associated with cardiovascular diseases, diabetes, hypertension, and sleep disorders [36–38]. Furthermore, diabetes is a powerful predictor of cardiovascular morbidity and mortality in patients. Diabetes-associated cardiovascular diseases arise by several complex mechanisms that are poorly characterized [37]. Moreover, a comparison of blood glucose and HbA1c between groups revealed significant differences, as expected. It was reported that the level of HbA1c and fasting blood glucose are two cardiometabolic risk factors in diabetic patients [39, 40].

In parallel with this, the significant differences of hyperlipidemia between the groups through the lipid profile evaluation have shown the pivotal impact of hyperlipidemia in patients diagnosed with diabetes along with CHD. Although diabetes and hyperlipidemia are two independent risk factors of CVDs, coexisting with them could increase the risk of CVDs [41–43]. Hopkins et al. [44] have reported more than 50% of patients diagnosed with CHD had familial hyperlipidemia. Moreover, in another research, it was revealed that familial hyperlipidemia was associated with severe early age coronary atherosclerosis. At the same time, there was a major decline in the rate of progression of coronary atherosclerosis when the levels of serum cholesterol are lowered by diet or drugs, resulting in the lowering of the risk for CHD [45].

In line with these, the categorical analysis revealed a significant association of smoking status and family history in CHD and coexisting groups without race interference compared to the control group. Several studies have shown that smoking is one of the leading causes of CVDs and could increase CHD risk [46–48]. Smoking could affect the cardiovascular system in several ways, such as: increases the heart rate; reduces the amount of oxygen transportation in the arteries; narrows the blood vessels, and consequently has a negative effect on the function of the heart and the cardiovascular system. Furthermore, smoking also causes endothelial dysfunction, leading to coronary spasms and consequently contributing to MI in patients. In parallel with this, several studies have reported the association between family history and the development of CHD at an early age. They have reported that the younger patients had more family history of CHD. They used age ≤ 55 and ≤ 65 years in men and women to define premature CHD in the family history, respectively. Furthermore, Garoufalis et al. [49] illustrated a positive family history of heart disease in 18% of young CHD patients. On the other hand, the race evaluation showed that despite the predominance of the number of ethnic Malays in experimental and controls group, there was no significant differences between subjects’ race in terms of the risk of CHD. However, Lee et al. reported a greater risk of CHD in the Indian males than the Chinese and Malay males. They even claimed three times greater risk of CHD in Indian ethnics compared to the Chinese [50]. Furthermore, in 1992, Rajadurai et al. indicated that the Indians below 50 years had a preponderance for CHD compared to other Asian races. However, they concluded that these ethic differences might be partly due to insulin resistance and the metabolic derangements resulting from it [51].

In this study, gene expression signatures in T2D, CHD, and coexisting conditions in Malaysian peripheral blood were evaluated using RT^2^ profiler™ PCR array, the latest technique, to determine whether T2D-related risk genes confer risk to CHD as well. In this regard, the coexisting dysregulated genes in phase one (T2D vs. control) and phase three (CHD + T2D vs. control) were selected, and the altered genes in phase two (CHD vs. control) were ignored. Phase 1 was conducted to determine the T2D risk genes in the Malaysian population. Previously, it was proved that the T2D risk genes were different in geographically distinct populations [52, 53]. Furthermore, phase two was conducted to subtract coexisting genes in phases one and two from phase one because these dysregulated genes were intrinsically CHD risk genes. Therefore, 11 common dysregulated genes were selected in phase one and three (T2D vs. control: CHD + T2D vs. control) which were the interested genes in T2D group that confer the risk to CHD. Given that the control group in both phases were the same, alteration in the T2D group and coexisting group illustrated that the mRNA level of AKT2, IL6, IL12B, IRS1, IRS2, MAPK14, and NFKB1 were higher in T2D + CHD patients compared to T2D patients. In contrast, the expression level of AQP2, FOXP3, G6PD, and PIK3R1 were lowered in T2D + CHD patients. The results of the current study showed that the expression level of AKT2 in patients diagnosed with T2D plus CHD was approximately 23 times higher than in the T2D alone group. The AKT2 is the most abundant AKT isoform in human insulin-sensitive cells and plays a pivotal role in insulin’s metabolic response, especially in type 2 diabetes mellitus and insulin resistance [54, 55]. Furthermore, in insulin resistance type 2 diabetes, AKT2 could be activated by insulin and consequently could inhibit insulin’s ability to stimulate glycogen synthesis [56, 57]. In this regard, the results revealed that the mRNA level of AKT2 in T2D patients was up-regulated 121 fold compared to the healthy group. Moreover, NF-κβ is one of the targets for AKT2 and could be activated by AKT2 as well. NF-κβ1 consists of proinflammatory properties and plays a key role in the pathogenesis of vascular complications of diabetes [58]. It was shown that hyperglycemic condition could activate NF-κβ1, which could trigger several transcriptions of a vast array of genes encoding proinflammatory cytokines such as IL1B, IL6, and IL12Bwas associated in the pathogenesis of atherosclerosis and consequently contributed to modulating the susceptibility to CHD [58–60]. Moreover, it was demonstrated that MAPK14 had a proinflammatory gene that was significantly up-regulated in type 2 diabetes mellitus and CHD [61–63]. The current study results have shown that the mRNA level of NF-κβ1, IL6, IL12B, and MAPK14 were up-regulated in patients with T2D alone and T2D plus CHD. Furthermore, the increasing ratio between two groups in these three upregulated genes was consistent as well. Moreover, Qi et al. illustrated that hyperinsulinemia causes myocardial insulin resistance and cellular dysfunction via IRS1 and IRS2 [64]. They inferred that the myocardial loss of IRS1 and IRS2 caused heart failure. In line with this, the insulin decreased mRNA expression of IRS1 and IRS2 in skeletal muscle of type 2 diabetic patients [65, 66]. However, the present study showed a significant elevation in mRNA expression of IRS1 and IRS2 in T2D and T2D plus CHD patients. This discrepancy between previous and present studies is not fully understood. However, it might be explained by the origin of the IRS1 and IRS2 which were collected for evaluation. In previous studies, heart, liver, and skeletal muscle cells were evaluated for genes expression. However, in the present study, the peripheral blood leukocytes were utilized for RNA extraction. In this regard, Jiménez-Navarro et al. revealed a significant and nonsignificant elevation in mRNA levels of IRS1 and AKT2, respectively, in circulating leukocytes of patients diagnosed with type 2 diabetes along with coronary defect [67]. In parallel, it was shown that PIK3R1 (p85α) expression was increased in type 2 diabetes and was associated with insulin resistance through PI 3-kinase mechanisms [68, 69]. Moreover, Zhao et al. illustrated the role of PIK3R1 as hub genes with 11 connections to other genes in CHD development [70]. Although the present study illustrated more than 1300-fold increment in PIK3R1 expression in coexisting groups, their expression was approximately five times lesser than the T2D group. In parallel with this finding, the current study has inferred that the expression level of AQP2, gatekeeper of water permeability, and FOXP3, a key player for the development and function of Treg (regulatory T cell), were drastically declined in T2D + CHD group around 57 and 170 times, respectively, compared to the patients in T2D group. In line with this, the expression level G6PD was highly up-regulated gene in both groups, however, in comparison between the two groups, G6PD slightly decreased in T2D plus CHD group compared to T2D only group. In this regard, Gupte et al. showed an elevation in the level of G6PD activity and protein expression in failing heart [71].

On the other hand, significantly dysregulated biological process/es and signaling pathway/s and consequently, the most presented genes in various biological functions and signaling pathways were identified using silico methods. Toward this end, the overlapped up-regulated genes were mainly enriched in glucose and insulin-related biological processes such as positive regulation of glucose import, glucose metabolic process, cellular response to insulin stimulus, insulin receptor signaling pathway, positive regulation of glucose metabolic process, positive regulation of glucose import in response to insulin stimulus, etc. which indicated that the mechanism for CHD progression in diabetic patients might relate to the dysfunction of the above biological process. Consequently, PIK3R1, IRS1, IRS2, and AKT2 were identified as the highly presented genes in the most significantly overrepresented GO terms. These findings revealed the pivotal role of diabetes in the progression of CHD in diabetic patients. In addition, the up-regulated genes were enriched toll-like receptor signaling pathway, insulin resistance, FOXO signaling pathway, TNF signaling pathway, etc., by KEGG pathway analysis.

Further investigation revealed that four genes (AKT2, PIK3R1, NFKB1, and MAPK14) are expressed in almost 50% of identified signaling pathways. By combining the highly presented genes that accumulated from GO terms and signaling pathways, six genes were identified as hub genes in these two terms. Moreover, Toll-like receptor signaling pathway was the most significant enriched signaling pathway through the overlapped genes in T2D and T2D plus CHD group. Toll-like receptor signaling pathway plays a vital role in innate immunity, inflammation, and immune cells regulation and survival. In the present study, perturbations of proinflammatory genes such as MAPK14, IL6, NFKB1, IL12B, etc., resulted in enriching the toll-like receptor signaling pathway [72].

Interestingly, previous studies have highlighted the role of chronic inflammation and activated innate immune mechanisms in the pathogenesis of T2D [73, 74]. It was reported that toll-like receptors (TLRs) contribute to insulin resistance and inflammation through activation of proinflammatory kinases. In addition, atherosclerosis is the underlying cause of CHD defined by the accumulation of lipids within the artery wall; however, it is currently inferred that atherosclerosis has a more complex inflammatory background [75, 76]. It was reported that the endothelial dysfunction and subsequent inflammatory response lead to chronic inflammation of the vessel wall, being the onset of atherosclerosis plaque formation [77, 78]. These findings confirm inflammation as a common link between T2D and CHD. Considering the role of the Toll-like receptor signaling pathway, authors believe that the immune system is a crucial component in the initiation and progression of CHD. Therefore, it was assumed that patients with diabetes present chronic inflammation, which potentially contributes to CHD development.

Consequently, further activation of inflammatory processes, in turn, could lead to other severe damage to coronary arteries. Activated immunity also may be the common antecedent of both T2D and CHD, which probably developed in patients with diabetes and, consequently, coronary atherosclerosis. Furthermore, it was demonstrated in the study by Liu et al. that the toll-like receptor signaling pathway interfered in the development of coronary artery stenosis and was associated with CHD severity [62]. Finally, to validate results, a single gene expression analysis of five randomly selected genes was conducted via Q-RT-PCR with increased and different sample sizes. All the target genes showed a significant dysregulation in the same direction with gene expression profiling with PCR Array.

## Conclusion

The sociodemographic factors analysis showed a significant correlation between weight, BMI, blood glucose, HbA1c, hyperlipidemia, smoking status, and family history with CHD progression without significant relationship with age and ethnicity. In the current study, 11 overlapped significantly up-regulated genes were identified and consequently suggested as potential markers that might be acting as CHD predictor genes in diabetic patients by comparing altered levels of genes expression between both groups to the control group. Furthermore, six genes (AKT2, IRS1, IRS2, PIK3R1, NFKB1, and MAPK14) out of 11 up-regulated genes were identified as hub genes enriched in most GO terms and signaling pathways. In addition, our results implied a role for inflammatory responses in the circulating leukocytes as a biomarker reflecting the initiation of atherosclerosis in a diabetic patient. These findings conclude that inflammation and immune-related genes such as IL6, NFKB1, MAPK14, FOXP3, and PIK3R1 are essential genes in CHD processes in T2D patients. Subsequently, these genes might be a significant target for treating and prevention these diseases. Therefore, this study has shown an association between T2D related genes and premature CHD development. However, further studies are needed to elucidate the role of the identified genes in the pathogenesis of CHD. Specific experiments must understand the true mechanisms of the signaling pathways in diabetic subjects that lead to CHD development.

## Data Availability

The datasets used and/or analysed during the current study are available from the corresponding author on reasonable request.

## Abbreviations

CHD: Coronary heart disease
T2D: Type 2 diabetes
CVDs: Cardiovascular diseases
CAD: Coronary artery disease
Q-RT-PCR: Real-time quantitative reverse transcription PCR
RNA: Ribonucleic acid
mRNA: Messenger ribonucleic acid
GO: Gene ontology
DAVID: Database for annotation, visualization, and integrated discovery
KEGG: Kyoto Encyclopedia of Genes and Genomes
IL12B: Interleukin-12 subunit beta
IL6: Interleukin 6
IRS: Insulin receptor substrate
MAPK14: Mitogen-activated protein kinase 14
NFKB1: Nuclear factor NF-kappa-B subunit 1
AQP2: Aquaporin 2
FOXP3: Forkhead box P3
G6PD: Glucose-6-phosphate dehydrogenase
PIK3R1: Phosphoinositide-3-kinase regulatory subunit 1
